# Supplementary material for: Multiomics Analysis of the Mechanism by Which Gibberellin Alleviates S-Metolachlor Toxicity in Rice Seedlings
Source: Plants (Basel). 2024 Sep 7;13(17):2517. doi: 10.3390/plants13172517 (PMC11396835; doi:10.3390/plants13172517)
Supplement: Supplementary file 1 [file plants-13-02517-s001.zip › plants-3129643-supplementary.pdf]

plants

MDPI

Supplemental materials

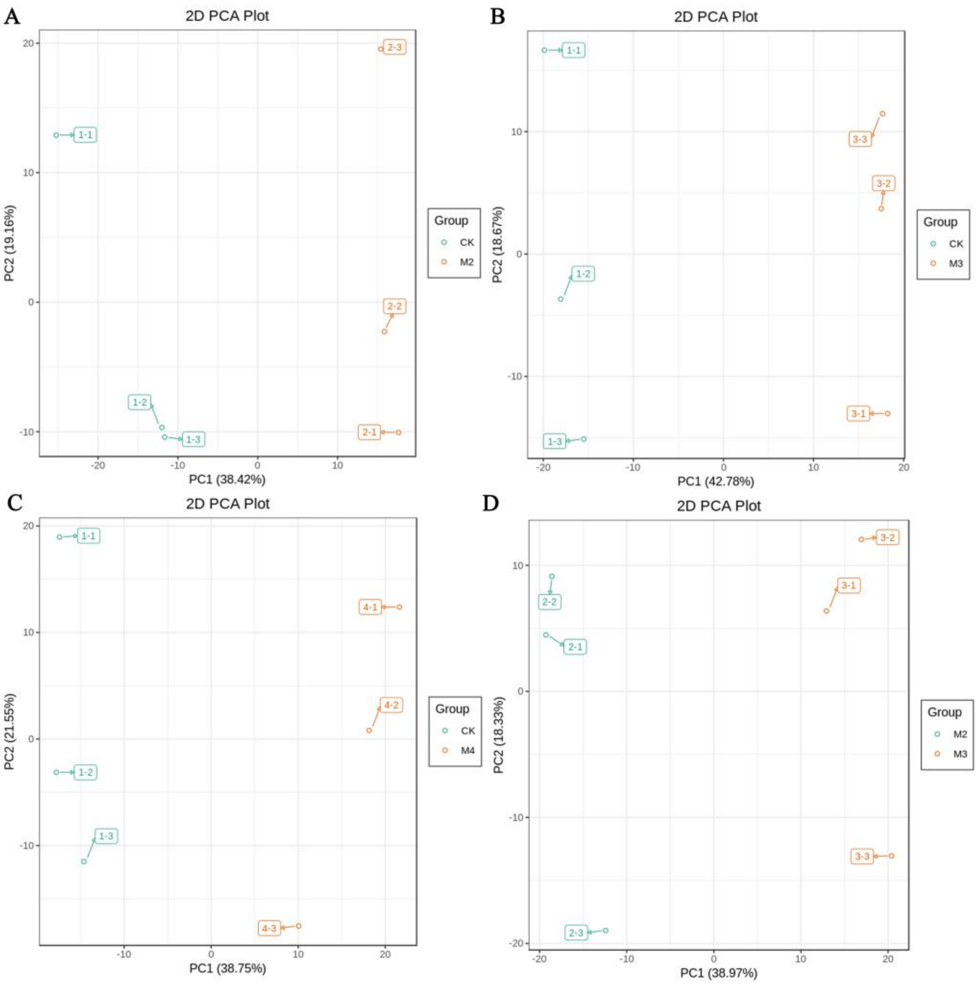

Figure S1: PCA analysis of metabolites. (A) CKvsM2 (B) CKvsM3 (C) CKvsM4 (D) M2vsM3. The X-axis represents the first principal component and the Y-axis represents the second principal component.

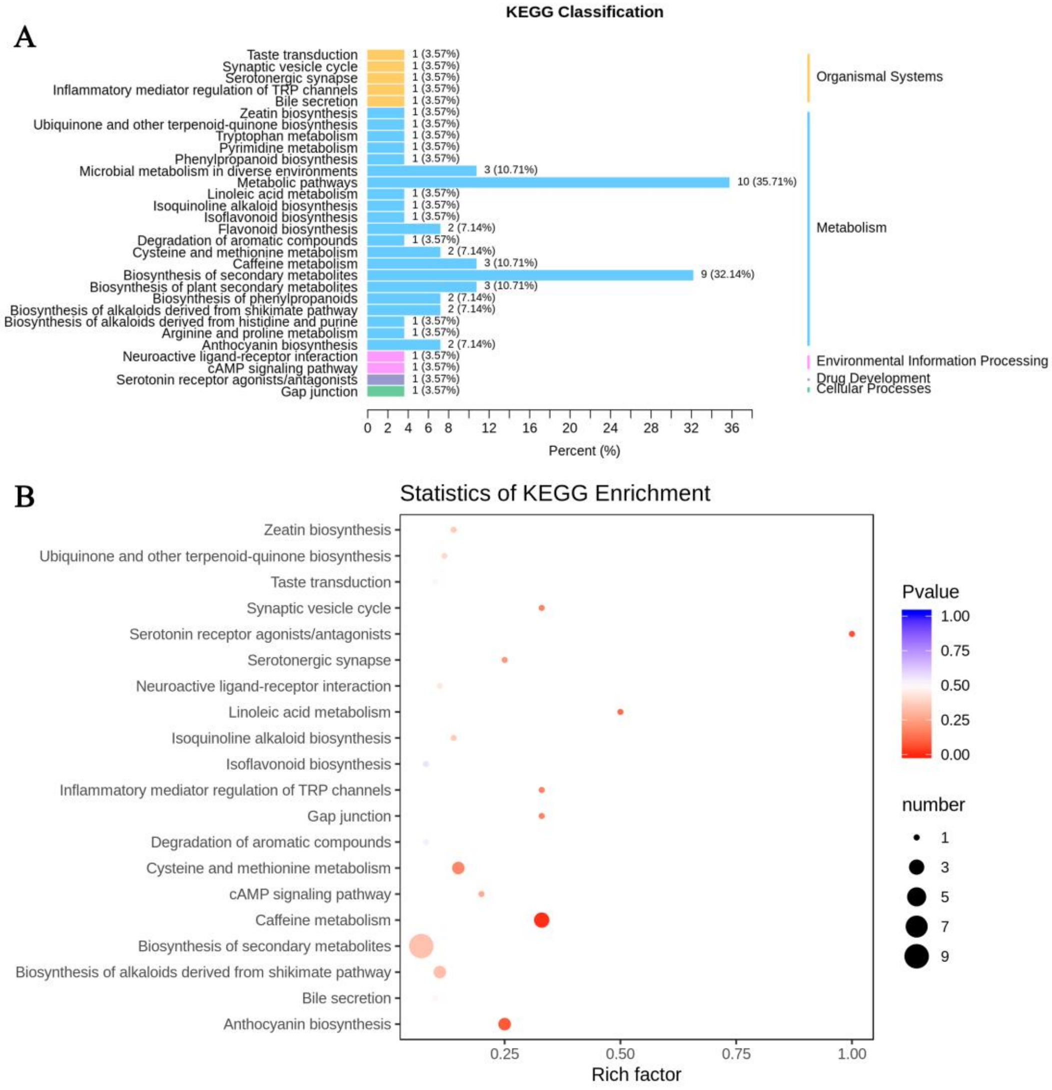

Figure S2: (A) The number of DEGs in different pathways (CKvsM4). DEGs are selected by annotated in pathway over 10. (B) bubble chart of KEGG pathways enrichment in CKvsM4. The control group was M2.

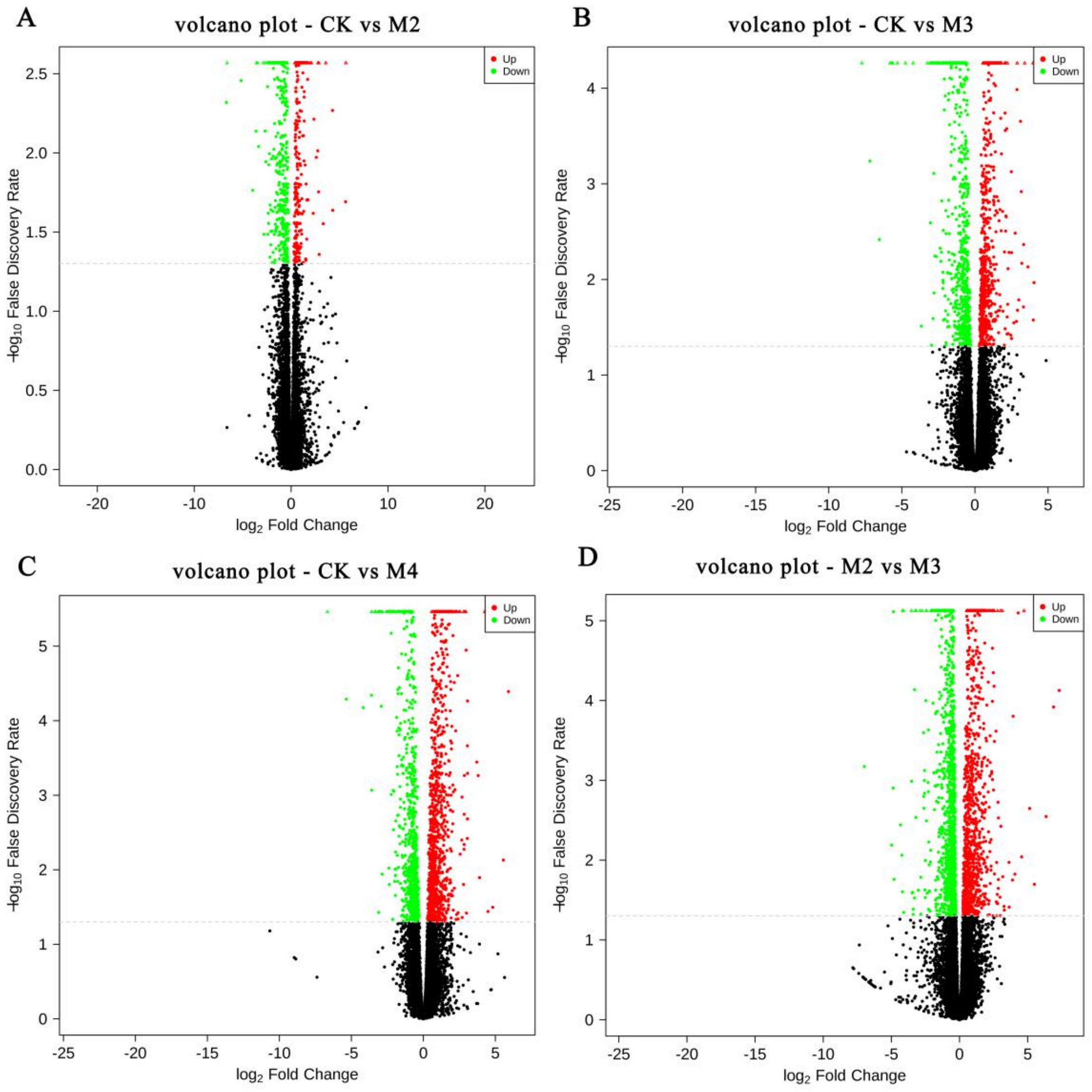

Figure S3: Volcano plot of DEGs. (A) CKvsM2 (B) CKvsM3 (C) CKvsM4 (The control group was CK) (D) M2vsM3 (The control group was M2). X-axis: Log_2FoldChange. Y-axis: -Log_{10} False discovery rate.

Table S1: Amino acid sequences of selected proteins

Protein ID	Sequence
A2WJQ8	MRAITGAVLSSKPCSLAKAARILDLFADSAASNLPSSDAATYLHTAADAT-<br>KNHHRFRLDLL-<br>NYYHRGDTASASDKKKRKRSEDHHQAATHVKQEQEAQQVAFAADLVAEEE<br>TDKETSKKNRKKKKHESQQENARDVKQNGGAPEQRFASPEKKRN-<br>KKNHPEEEEV-<br>KTVVKGIIVSAGDSVATEKKRKKKRERADDTDNDKEQVEHTRKKPRNRS*

Protein ID	Sequence
A2WJR3	MAAQSSTPSPQDGSGRGEFSDNLVLQTPQPMREDYIQNAVNFLGHPKVKG-<br>SPVFYRRSFLEKKGLTKEEIDEAFRRVPDPQPNSTDVAAVASQQAGIANQSAG<br>VQPYETVQAPQAINTGPIVPHAQPQLSWSRTLIGVGVFLGVGASAA-<br>VILKKLFVPRLKSWIQGAHVEGDEISGNELKSKFYEEIKAAIQDSASAFSDIAK<br>TNQELLASKDEDKKILTKLAQAFDSQAEAFRSLSDSLNRM-<br>SENRFYQYNLMEDHFQSAP-<br>WNGPTTNSWRASQQTNAYNTSPRSDFDSGRHPFMPVPGEPSPGAFPARSYVE<br>QQRMQRPGYGFQPQMSNDRWNPGSPLTNYHGAP-<br>PYQQYHHGSTNAIDEAPAPAPVPAPAP-<br>PAESPFQRRWVPPQPPGVVMPEAVAAIRQPRQQVAAASRPSESAAATEQPQS<br>GDVAGGAAMANAGNGEAEQEREAAA*
A2WL79	MAISEMSALFFLFSALLRSSLVHSQGLQIGFYDNNCPDAE-<br>DIVRSTVEKYYNNDATIAPGLLRLHFHDCFVQGCDASVLISGASSERTAPQNF<br>GIRGFEVIDDAKSQLEAVCSGVVSCADILALAARDAVDLTGG-<br>PSWSVPLGRRDGRISSASDA-<br>KALPSPADPVSVQRQKFAAQGLTDRAHTIGQTDCIFFRYRLYNFTATGNADPT<br>ISPSALPQLRALCPPAGDGSRRVALDLG-<br>SPGAFDVSFFKNVRDGGAVLESDQRLWG-<br>DAATQAAVQSFAGNVRGLFGLRFSYEFPKAMVRMSSIAVKTGSQGEIRRKCSK<br>FN*
A2WMB2	MELSSLAALLHSPLLLAVLLLVFSWLIVSSTKKRPPPPCGDGGRRLPLPP-<br>SPPGVPLLGHLPLLGTLPHRKLRSMAEAHGPVMLLRLGRVPAVVASSAAAAE<br>EVMRTRDLAFASRPRVRMSERLFYGRDMAFAPYGEFWRQARRVTVLHLL-<br>SPRRVLS-<br>FRGVREQEVAALLDRVRRRCGGGGETVNLSDLLMSYAHGVISRAAFGHGGA<br>HGFDGDEGGEKLRKLFADFEGLLGTMTVGEFVPW-<br>LAWVDKLTGLDAKVARTSAAMDGLLER-<br>VIADHRERRRSRGQAVGDGEADADHRDFVDVMLDVSEAEEGAGAGAGGVL<br>FDTVAIKAVILDMMAAGTDSSFTTTEWVMAELINHPRVMRKLQDEIRAV-<br>VGTSSASAAAAATGGGQVTEDHLGELPFLRAVIKEMLRLHAPGPLLLPRETV<br>EDTELLGYRIPARTRVIINVWAIGRDAAAWGDSAEEFVPERWLD-<br>GGGGGGVEYA-<br>QQLGKDSRFVPFGAGRRGCPGAGFAALSVELALANLLYHFDWELPPPAASGI<br>MATTRLDMDELFGLSVRLKADLNLVAKPWSPGAS*
A2WMB5	MHAHKRRCATMAVSLVVVVVVVVIAIVVPLLYLVLLPAWK-<br>PARRDDGDGGMRRRLPP-<br>SPPWGLPLLGHLHLLGALPHRALRSLAAAHGPVLLLRLGRVPVVVVSSAAA<br>AEEVMRTRDLEFASRPRVAMAERLLYGGRDVAFAPYGEYWRQTR-<br>RICVVHLLSARRVLS-<br>FRRVREEEAAALVARVRAAGGAVDLVEHLTAYSNTVVSRAVFGDESARGLYG

Protein ID	Sequence
	DVDRGRVLRKLFDDFVELLGQEPMGELLPWLGWVDALNGMEVKVQRT-<br>FEALD-<br>GILEKVIDDHRRRRREVGRQMDDGGGGDHRDFVDVLLDVNETDMDAGVQ<br>LGTIEIKAIILDMFAAGTDTTTTVIEWAMAELITHPDAMRNAQDEIKAV-<br>VGITSHITEDHLDRLPYLKAVLKETLRLHPPLPLLVPHEPSSDTKILGYSIPACTR<br>IVINAWTIGRDQATWGEHAEEFIPERFLESGLDYIGQDFVLVPF-<br>GAGRRGCPGVGFAVQAME-<br>MALASLLYNFDWETRVVDRRSEFGTSSLDMSEMNGLSVRLKYGLPLIAISRFP*
A2WN51	MGCLLMLCLVSPLLLATSVHGNPWYGYGYGLFPQFYDHSCPKA-<br>KEIVQSIVAQAVARETRMAASLVRLHFHDCFVKGCDASVLLDNSTTIISEKGS<br>NPNMNSLRGFEVVDEIKAALEAACPGTVSCADILALAARDSTVLVGGPY-<br>WDVPLGRRDSL-<br>GASIQGSNNDIPAPNNTLPTIITKFKRQGLNIVDVVALSGGHTIGMSRCTSFRQ<br>RLYNQSGNGMADYTLDVSYAAQLRQGCPRSGGDNNLFPLDFVSPAK-<br>FDNFYFKNILSGKGLLSSDQVLLTKSAETAALVKAYADDVNLFFKHFAQSMV<br>NMGNISPLTGSQGEIRKNCRRLNNYYH*
A2WNB5	MDLAWWFAVAVVVCGLVGGG-<br>SAGLLETNPGLAYNFYQKSCPNVDSIVRSVTWAQVAANPALPGRLLRLHFHD<br>CFVQGCDASILLDNAGSEKTAGPNLSVGGYEVIDAIKTQLEQAC-<br>PGVVSCADIVA-<br>LAARDAVSYQFKASLWQVETGRRDGPVSLASNTGALPSPFAGFSTLLQSFANR<br>GLNLTDLVALSGAHTIGKASCSSVTPRLYQGNTTSLDPLLDSAYA-<br>KALMSSCPNPSPSSS-<br>TIDLDVATPLKFDSGYYANLQKKQGALASDAALTQNAAAAQMVADLTNPIK<br>FYAAFSMSMKKMGRIDVLTGSKGNIRKQCRSAS*
A2WNR8	MRLSVAILCALVAVQAAALLLAGSAAAASELKVGYYHKKCKGVENVIKWH-<br>VIKALKQNRRT-<br>GAALVRLLFHDCFVRGCDGSVLLDKSYENPHPEKEAPVNIGLAAFDLLEEIK<br>AAVEKRCPGVVSCSDILIYAARDAGSILSNGHVHFDVPAGRLD-<br>GVVSRADEAQAELPDSTMTVQQLKDNFAAKGFDTEQLVILSGAHSIGQGHC<br>SSFTGRLSEPPQQITPAYRDLLNYKCSQAANPDVVNNVRD-<br>EDASVVARFMPGFVSRVRKISDFLDNTYYHNNLAKIVTFHSDWQLLTDATSLS<br>KVHEYADNATLWDSDFSDSLLKLSQLPMPEGSKGEIRKKCSAINHLY*
A2WPA3	MASCSKWLAGLMLLAAALACSLPAASRAQLQVGFYNTSCPTAEALVRQAV-<br>VAAVANNSGLAA-<br>GLIRLHFHDCFVRGCDASVLIFSPNGTAERDAAPNNPSLRGFEVIDAAKAAVE<br>AACPRTVSCADILAFAARDSVNLTGNSFYQVPAGRRDGNVSIDTDAFT-<br>LPGPNL-<br>TATQLVDGFKLRNLTAEEMVILSGSHTIGRSHCASFLFKNRERLANGTISPAYQ

Protein ID	Sequence
	ALLEALCPPTTGRFTPITTEIDVSTPATLDNNYYKLLPLNLGLHFSDDQLIR-<br>NATLL-<br>PFVDAFAANETLWKEKFVAAMIKMGNIDVLTGARGEIRLNCSAVNPSSSSSSS<br>SAGRMIETVFPGAGGEVAAS*
A2WPA9	MAMKCLFLFFAFLVAFFPGAAVGAGLKVGFYNKTCPSAERLVQQAVAAAF-<br>KNNSGVAPGLIRLHFHDCFVRGCDASVLIDGNDTEKTAPPNNPSLRGFEVID<br>AAKAAVEAACPRVVSCADILAFAARDSVALTGNVTYKVPAGRRDGNVSIA-<br>QDALDNLPPPTF-<br>NATELVGRFANKSLTAEDMVVLSGAHTIGVSHCDSFTSRLYNFTGVGDADPAI<br>SAAYAFLLRAVCPSNSSQFFPNTTVDMDVITPAALD-<br>NKYYVGVANNLGLFTSDHALLTNATLRASVDEFVKSETRWKSKFVKAMVKM<br>GGIEVKTGTTQGEVRLNCRVVNKRSANAELELELAAAMDDGDEVAAS*
A2WR13	MDFELRKAREKLEREQRERVQRAKDKADRERRAKAE-<br>AARRRDALEASHRERRLDAAR-<br>AQEEAQQKMEEVMQLGKGISFLHMFEALRYDGPGDKIKLPPSSFKELSDEGA<br>LDKGPMYFRLSKVRDSVPGAPQDNDADEATCCGVLEFTAREGSAELT-<br>PHVWNNLFRGD-<br>SPDVPLIEVRYVSLPKGTYAKLKPEGVGFSDLPNHRAVLETALRNHATLSEND<br>FVVVNYGQLQYKLKVLELKPASSVSVLETDVEVDIEGPDSVLD-<br>NVENQHVLVPLETGKVESGVVEEGKFRYYKFLVDEGMGEKVASRHANIEVKI<br>ETYTSGGDTDIYVSRHPLVFPTQHRHEWSSHEMGSKVLILKPR-<br>DASLSSGTYSIGVYGFKGTTKYQLSVAIKDVLNGQRIGEQASASSSVDVDSVVC<br>KNCKRYISNRTSLLHEAYCVRHNVVCMHDGCGVVLR-<br>KEEATDHVHCNKCGQAFQQREMEKHMKVFHEPLQCPCGVVLEKEDMVQH<br>QSSTCPLRLIVCRFCGDTVQAGGEPLDARDRLRNMCEHESICGS-<br>RTAPCDSCGRSVMLKDMDIHVIAVHQKS*
A2WRL7	MFFQRKNSKKVKDSNGSSSKKDKDSRGKNIFDSAKGGLGALTGTLQTAK-<br>NDAGEKAENLQDDVKTTIGAILRRGSGVLEKAKEIGGHSEASQSKELEQGSEE<br>QGKKDMEALTKVMDKVKEVKSNPEVVEKLDKVKE-<br>DISSLAHALHLGKHDKEHESEEKAKEGE-<br>TAKRADEGASASKSEDSGVVVQAVEEIQAVVTAVQQQLHTEGAAAETPNEA<br>AAAETSAEGEKPEESKRDVEKDDPSKRLDFKGFFAMIFERCCNPGNKKKD*
A2WTQ2	MAPVAVGDTLPDGQLGWFDGEDKLQQVSVHGLAAGKKVVLFGVP-<br>GAFTPTCSNQHVPG-<br>FINQAEQLKAKGVDDILLVSVNDPFVMKAWAKSYPENKHVKFLADGLGTYT<br>KALGLELDLSEKGLGIRSRRFALLADNLKVTVANIEEGGQFTISGAEEILKAL*
A2WXN0	MWSAAAARTVT-<br>PLPAASPLQQHQQRRGAWARVGNGRAASTTAARAGLWDFVGG-<br>DLVKPDMGRWLDDVEKHKSLAIYPPHEGGYEGRYLSRLSYQGYYFLDLSARG

Protein ID	Sequence
	LGDPETTLTKIHPVCPPS-<br>LGRQPVARWYFPPEVDYRLSLLHPDAKGLVVWVMEAKVLSKAEL-<br>QFLAILPDIRPKVRVIAECGNWRKFVWKPLKQIAGLEPDPDAEE*
A2WZD6	MASRRSAAASPAMAAAAAVVAVSVYLLVCSAPAAATAAA-<br>VAKKPSYPPVAKGLSFDHYK-<br>KSCPQAEAIVFSFLRDAIGKDVGLAAALIRLHFHDCFVQGCDASILLTKTPGG<br>PDGEQQAIPNESLRPAAFKAVNDIRALLDRAC-<br>GRVVSCSDIVTLAARDSVKLAGGPSYKVPLGRRDGLTSATPSQVLGALPPPTS<br>HVPELIAALAKLNLDAADLIALSGAHTVGIAHCTSFT-<br>GRLYPKQDGTMDKWFAGQLKLTCP-<br>KNDTANTTVNDIRTPNAFDNKYYVDLQNRQGLFTSDQDLFVNATTRPLVAE<br>FAVDQSAFFHQFVFSVVKMGQIQVLTGSQGQIRANCSVRNPGAA-<br>SADEELPWSAAVETVVEAAESIVL*
A2WZD9	MASRTSATAGMLLLAAAAALVCS-<br>SAAARMPPLAKGLSLGYYDASCPQAEAVVFEFLQDAI-<br>AKDVGLAAALIRLHFHDCFVQGCDASILLDSTPTEKSEKLAPPNKTLRKSAFD<br>AIDDLRDLLDRECGDTVVSCSDIVTLAARDSVLLAGGPWYDVPLGRHDGSS-<br>FASEDAVLSALPSPDSNVTTLLEALGKLKLDAHDLVALSGAHTVGIAHCTSFD<br>KRLFPQVDPTMDKWFAGHLKVTCPVLNT-<br>NDTTVNDIRTPNTFDNKYYVDLQNRQGLFTSDQGLFFNATTKPIVTKFAVDQ<br>SAFFDQYVYSVVKMGMIEVLTGSQGQIRKRCSVSNAAAAGDRAWSVVET-<br>VAEAAESLVL*
A2X1X4	MAAPTAAALSTLSTASVTSGKRFITSSFSLSFSSRPLATGVRAA-<br>GARAARRSAASASTVVATIAVGDKLPDATLSYFDPADGELKTVTVAELTAGRK<br>AVLFAVPGAFTPTCSQKHLPGFIEKAGEL-<br>HAKGVDAIACVSVNDAFVMRAWKESLGLG-<br>DADVLLLSDGNLELTRALGVEMDLSDKPMGLGVRSRRYALLADDGVVKVLN<br>LEEGGAFTTSSAEEMLKAL*
A2X268	MNRKPGDWDCRACQHLNFSRRDLCQRCGEPRGAADRGSGGGGDYANFG-<br>GRGGSS-<br>FGGGFGTGSDVRPGDWYCNCGAHNFASRSSCFKCAAFKDDAAVNSGGAGA<br>FDGGDMSRSRGYGFGSGAV-<br>RASRPGWKSGDWICTRSGCNEHNFASRMECFRCNAPRDSGSAMTYENYLH*
A2X827	MEKGERSERLFYKLLIDNVEELLPVVYTPTVGEACQKYG-<br>SIYRRPQGLYISLKDKG-<br>KILEVLKNWPERSIQVIVITDGERILGLGDLGCQGMGIPVGKLSLYTALGGVRA<br>PCLHLHPSATIIAAITSVLAPSLTHRFPLRFALSPLRVASLHPHRIAPLIIPSWV-<br>TSFHIAASSGFVCSRPLADHP*
A2XEA4	MGAVAAVRAAVLVVAVALAAAAAGASAQLCDKYYD-<br>GTCPDVHRIVRRVLKRARQDDPRIFASLTRLHFHDCFVQGCDASILLDNSTSIV

Protein ID	Sequence
	SEKFATPNNNSARGYPVVDDIKAALEEACPGVVSCADILAIAAKISVELSGG-PR-WRVPLGRRDGTTANLTGADNNLPSPRDNLTTLQQKFAAVGLDVTDLVALSGAHTFGRVQCQFVTDRLYNFSGTGKPDPTLDAGYRRALAKSCPRRGGNSSAL-NDLDPTTPDAFDKNYFANIEVNRGFLQSDQELLSTPGAPTAAIVNSFAISQKAFFKSFARSMVNMGNIQPLTGSQGEVRKSCRFVNGS*
